# Effects of Building Orientation and Raster Angle on the Mechanical Properties of Selected Materials Used in FFF Techniques

**DOI:** 10.3390/ma17246076

**Published:** 2024-12-12

**Authors:** Piotr Dziewit, Kamil Rajkowski, Paweł Płatek

**Affiliations:** Faculty of Mechatronics, Armament and Aerospace, Military University of Technology, 2 Gen. S. Kaliskiego Street, 00-908 Warsaw, Poland; kamil.rajkowski@wat.edu.pl (K.R.); pawel.platek@wat.edu.pl (P.P.)

**Keywords:** PLA, ABS, Mediflex, additive manufacturing, Charpy impact test, tensile test, FFF, raster angle, building orientation, fractography

## Abstract

Advances in the development of additive manufacturing materials (AM) and the low availability of studies on the impact response of AM specimens are the main reasons for this paper. Therefore, the influence of building orientation (vertical and horizontal) and the angle of the raster (15°/−75°, 30°/−60°, 45°/−45°, and 0°/90°) on the tensile and impact strength of AM specimens was investigated. The polylactic acid (PLA)-PolyMax, Mediflex and acrylonitrile-butadiene-styrene (ABS) filaments were chosen to provide a comprehensive characterization of AM materials with versatile mechanical properties. The experimental results of this study show that the tensile strength and toughness of PolyMax PLA specimens are comparable to ABS specimens, while Mediflex samples are characterized by their higher toughness, but lower impact force needed to break the samples. The Mediflex Charpy fracture surfaces exhibit a ductile character compared to those of brittle ABS and PLA. Furthermore, fracture surface morphology shows the allocation of voids, which helps us to understand differences in mechanical properties, and allows one to properly interpret the results of the geometrical accuracy of AM specimens with various printing settings.

## 1. Introduction

Additive manufacturing (AM) was developed in the industry as a promising technology that allows for the prototyping and production of three-dimensional (3D) parts [1,2,3]. Compared to conventional methods, the advantages of this manufacturing technique include the design freedom that it offers, a reduction in the price of prototype models, and a significant minimization in the time needed from the design to production stages of the real models [4]. Furthermore, AM enables the reduction of material wastage, especially when objects with complex shapes are built. The abovementioned features have caused AM to be successfully introduced and commonly used in many advanced branches of industry, such as automotive, aerospace, defence, bioengineering, medicine, sports, and civil engineering [3]. One of the most popular groups of AM techniques includes fused deposition modelling (FDM) and fused filament fabrication (FFF) methods [5,6], sometimes named interchangeably. Both are based on the extrusion of the thermoplastic materials through the nozzle onto the base plate [7]. They allow for the manufacturing of objects made from various types of thermoplastic polymers characterized by different mechanical and physical properties. Moreover, currently, they enable building parts made from composite materials where continuous or cut fibres (carbon, aramid, Kevlar) are used to improve mechanical strength [8,9,10]. This group of 3D printing techniques is well-known and commonly used [6].

Despite its many advantages, such as the low cost of manufacturing, the wide variety of 3D printers with diverse technological capabilities [11], and the broad range of filament materials with different physical and mechanical properties, the FFF/FDM technique also has some drawbacks [12]. One of them is the limited mechanical strength resulting from the specific layer-by-layer building method, which is highlighted in many research papers [12,13,14,15]. To improve the mechanical integrity of fabricated structural components, an approach that utilizes full in-fill is often adopted [16]. In this technique, material is deposited in consecutive layers along adjacent parallel paths. To counteract the inherent anisotropic mechanical characteristics of the fabricated object, it is common to vary the orientation of these layers. A widely adopted strategy for adjusting the orientation of successive layers is to set them at a 45-degree angle relative to each other. This orientation adjustment aims to optimize the mechanical properties across different directions and enhance the overall robustness of the component. This 3D printing technique aims to achieve isotropic mechanical properties for an object when it is subjected to external loading, such as compression or tension, in the XY plane relative to the object’s fabrication direction along the Z-axis. By carefully aligning the deposition of materials in varying orientations, the method seeks to balance mechanical properties across different axes, thereby enhancing the object’s overall mechanical integrity and performance under applied loads in the specified plane.

Nevertheless, changing the layer fill angle can adversely affect the surface roughness of components produced using fused filament fabrication (FFF) 3D printing technology. Therefore, it is crucial to determine the optimal layer fill angle that strikes a balance between the high surface quality of the produced parts and their mechanical strength.

Due to this reason, to find a compromise between satisfying surface roughness and high mechanical properties, various types of model fillings are used.

Furthermore, the mechanical properties provided by the filament producers are often limited to specific 3D printing conditions. These problems attract the attention of many researchers. It is well known knowledge that setting the raster angle to −45°/45 helps distribute stress uniformly. Dawoud et al. [17], as well as many other scientists [18,19,20] reported that a raster angle of 45/−45° for the acrylonitrile-butadiene-styrene (ABS) parts manufactured with FDM can result in higher mechanical and fracture resistance, as well as static and fatigue performance. The same conclusions have been drawn by other authors [21,22], where polylactic acid (PLA) material was investigated. Nevertheless, Khosravani et al. [23], who studied the influence of raster layup and printing speed on the mechanical strength of material samples made from PLA, found that mechanical properties like stiffness and strength strongly depend on the raster angle where the highest and the lowest strengths were obtained for 0° and 90°. A similar investigation was undertaken by Qayyum et al. [24] where authors focused their attention on the relationship between raster angles as well as infill patterns on an in-plane and the edgewise flexural properties of ABS material. Based on the results of experimental studies, they stated that the raster angle strongly determined the mechanical behaviour of material fabricated via 3D printing. The value of flexural strength registered for samples where the raster angle was 0° was approximately two times higher in comparison to results gathered for samples where the raster angle was 90°. Furthermore, the authors drew the conclusion that materials manufactured additively via the FFF technique indicate a strong anisotropy. Due to this reason, they recommended further studies in this research area [24]. Similar conclusions were formulated in the following works [7,25,26].

Based on the conducted literature review, it was found that the research conducted by scientists on the influence of the infill angle of individual material layers has a limited scope [27]. Most often, they are limited to one type of material and an infill angle range of 0, 45, and 90 degrees. Moreover, the literature contains results of mechanical properties research mainly related to tensile strength tests. To supplement the knowledge in this area, the authors of the paper decided to expand the scope of the conducted material tests, including additional infill variants in the tests (15°/−75°, 30°/−60°, 45°/−45°, and 0°/90°). Additionally, the tests were carried out for two standard materials, PLA and ABS, that are commonly used in FFF additive manufacturing techniques, and for an additional material variant, Mediflex, which is characterized by a very large range of plastic deformation that can be used in medical applications [28]. The assessment of mechanical properties was conducted not only based on static tensile testing, but also included impact testing.

## 2. Materials and Methods

Three types of thermoplastic materials commonly used in FFF 3D printing techniques were selected for experimental research. Among the wide range of available options, PLA, ABS, and Mediflex were chosen. PLA is one of the most frequently chosen materials due to its high technological versatility, low impact of thermal shrinkage on the deformation of produced objects, low cost, and wide range of available variants. On the other hand, ABS is characterized by a higher mechanical strength and greater resistance to dynamic loading than PLA. Unfortunately, it is prone to thermal shrinkage and requires stable temperature during the 3D printing process.

Mediflex is purported to serve as a material bridging the gap between brittle and ductile materials. Mediflex blends seamlessly with ABS, allowing for the creation of composite materials. It indicates a thermal resistance up to 120 °C. The filament is slightly harder than typical flexible materials, with a hardness of approximately 96–98 Shore A. Consequently, the authors selected materials listed in Table 1, along with their respective properties, for comprehensive evaluation and comparison.

The FFF 3D printing process was carried out using a Prusa MK3S+ 3D printer (Prusa Research a.s., Prague, Czech Republic) for all materials enumerated in Table 1. This printer features a single nozzle printing head equipped with a direct feeding mechanism, specifically designed for handling flexible materials like Mediflex. The materials utilized for this 3D printer are supplied in a filament form with a diameter of 1.75 mm and wound onto a spool. Throughout the printing procedure, the material is extruded through the nozzle onto a heated build plate. The build plate is constructed with a magnetic steel textured bedplate, complemented by a polyetherimide (PEI) surface to facilitate the adhesion and fixation of the samples. The nozzle assembly moves along the 0ZX axes, while the build plate traverses along the 0Y axis, ensuring precise layer deposition and uniform build quality.

Initially, technological tests were conducted to determine the optimal 3D printing parameters for the selected filaments using a Prusa i3 Mk3S+ 3D printer. The results of these tests led to the identification of 3D printing parameters listed in Table 2. The specified parameters deviate slightly from the manufacturers’ recommended settings for each filament type.

**Figure 1:**
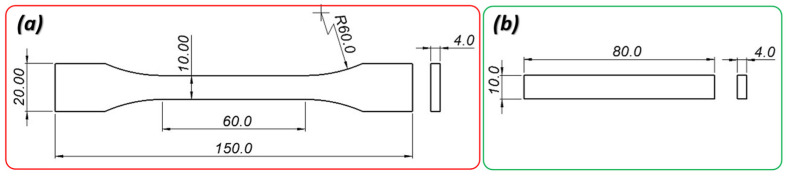
Dimensions of specimens: (**a**) dog-bone specimen for tensile test; (**b**) unnotched specimen for Charpy impact tests.

Subsequently, material samples were fabricated for mechanical property testing. The authors proposed an assessment of mechanical properties based on the results of static tensile tests and impact resistance tests. The study employed solid material samples with dimensions and shapes conforming to ISO 527-1 [29] and ISO 179-1 [30] standards (Figure 1).

**Figure 2:**
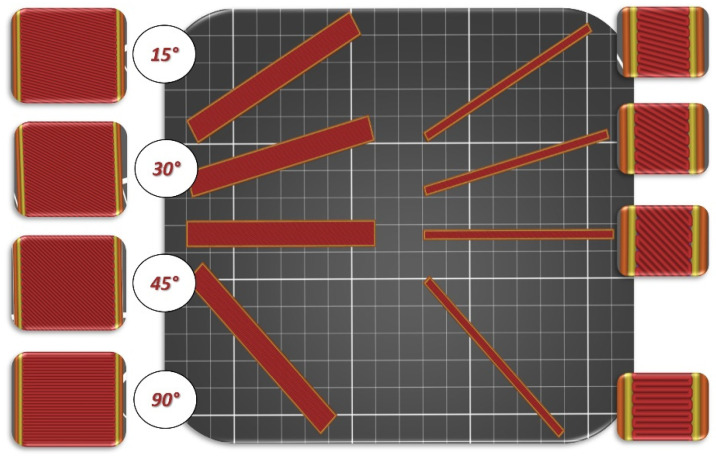
Infill pattern and orientation of the samples on the printing bed: (**right side**) horizontally and (**left side**) vertically oriented specimen for Charpy impact test; with different raster angles: 15°/−75°, 30°/−60°, 45°/−45° and 0°/−90.

To evaluate the influence of the infill angle on the samples, it was decided to produce them using the following angular infill patterns: 15°/−75°, 30°/−60°, 45°/−45°, and 0°/90°, designated hereafter in the article as respectively: 15°, 30°, 45°, and 90°. Additionally, impact resistance test samples were fabricated considering two manufacturing orientations, horizontally and vertically, as illustrated in Figure 2.

**Figure 3:**
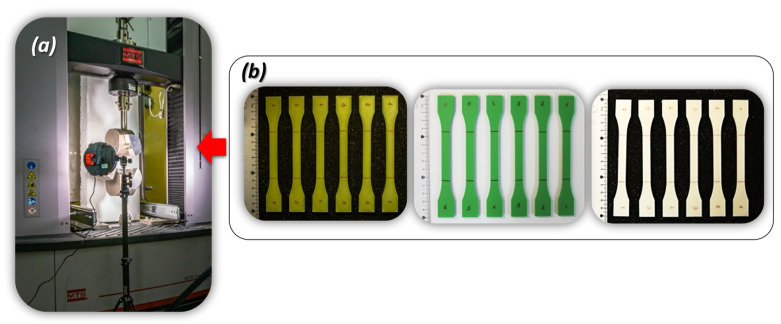
The main view of the laboratory stand used to perform quasi-static tensile tests: (**a**) MTS Criterion 45.105 strength machine with additional lightening system, (**b**) dog-bone specimens made from PLA PolyMax, ABS+ and Mediflex filaments.

The tensile mechanical properties of the polymer materials were evaluated using an MTS Criterion 45.105 testing machine (Figure 3a), following the ISO 527-1 [29] standard for uniaxial tensile testing. Tensile specimens, as depicted in Figure 3b, were subjected to a strain rate of 0.01 s^−1^. Throughout the tensile testing procedure, the TW-Elite MTS TestSuite 2.3.1 software was employed to monitor and record the entire process. Additionally, a high-resolution camera was utilized to capture the tension behaviour of the specimens.

Experimental data were acquired at a sampling frequency of 50 Hz, enabling the generation of precise stress-strain curves. These tests were conducted to ascertain and compare the mechanical strength properties and failure mechanisms of samples fabricated with different variants of material infill.

**Figure 4:**
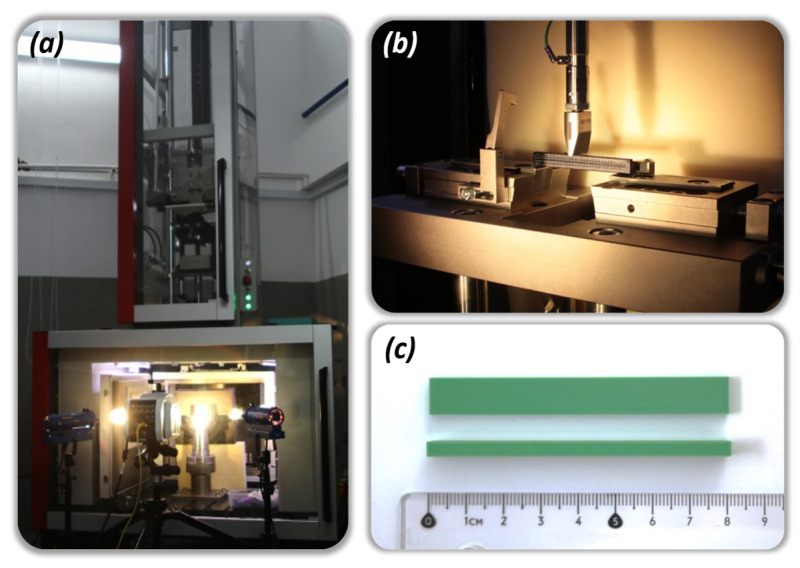
The main view of the laboratory stand used to perform Charpy tests: (**a**) Zwick/Roell Amsler HIT2000F with fast camera Phantom v1612, (**b**) sample support with impactor, (**c**) exemplary sample made from PLA filament.

Impact tests were carried out using the Zwick/Roell Amsler HIT2000F drop tower following the ISO 179-1 [30] standard (Figure 4). The tests were executed under ambient room temperature conditions, with a striker mass of 9.336 kg and speed set to precisely 2.9 mm/s at the point of impact. Prior research by Graupner et al. [31] delved into the intricacies of specimen geometry, specifically exploring the impact of notches on sample behaviour. Their findings highlighted a nuanced sensitivity to notches, varying based on the angle of the notch and the absence or presence of notches altogether. Furthermore, the layered nature inherent in the manufacturing process can introduce geometric distortions to notches, potentially affecting test outcomes. In light of these considerations, this study employed unnotched specimens with dimensions of 80 × 10 × 4 mm (as illustrated in Figure 1b), oriented for edgewise impact. The selected test conditions were tailored to account for the unique wall structure of the specimen relative to its infill pattern, recognizing the potential impact of these design intricacies on test results.

The mechanical characteristics of various sample configurations were evaluated through rigorous testing procedures, encompassing three distinct material types. A comprehensive total of 72 tensile tests and 144 impact tests were conducted to ascertain these properties.

## 3. Results

**Figure 5:**
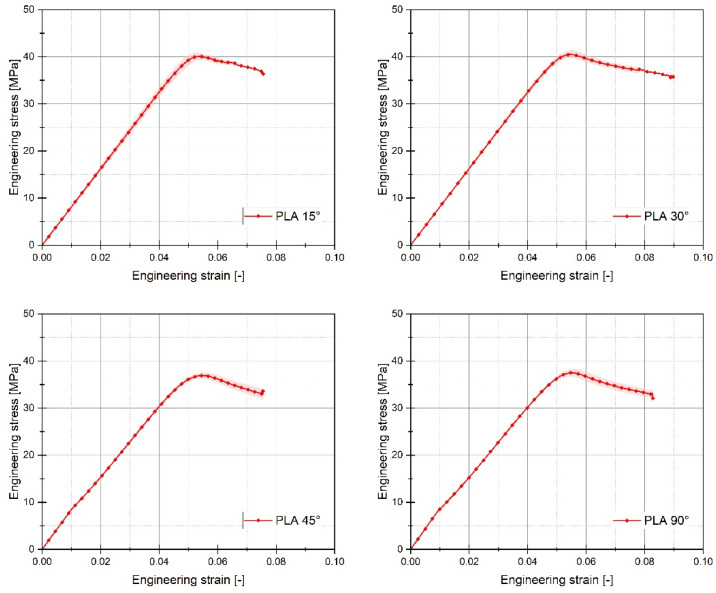
Stress-strain plots registered for 3D printed PLA PolyMax filament with consideration of different raster angles: 15°, 30°, 45°, and 90°.

**Figure 6:**
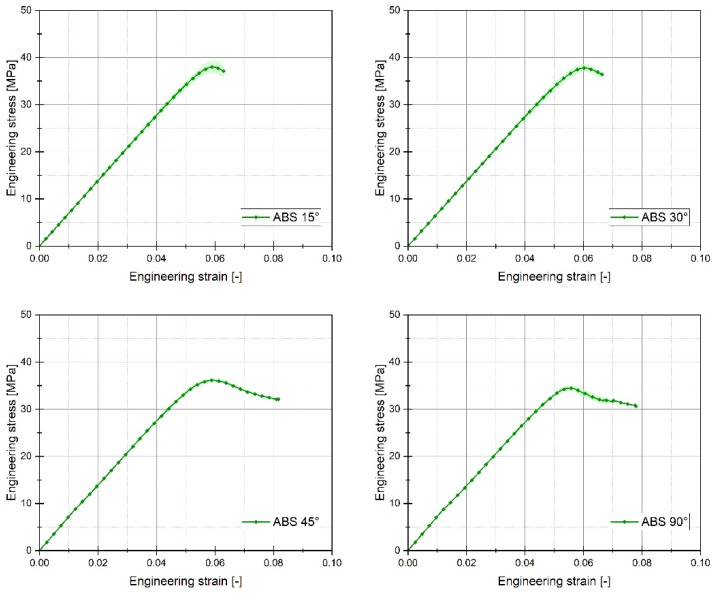
Stress-strain plots registered for 3D printed ABS Plus filament with consideration of different raster angles: 15°, 30°, 45°, and 90°.

**Figure 7:**
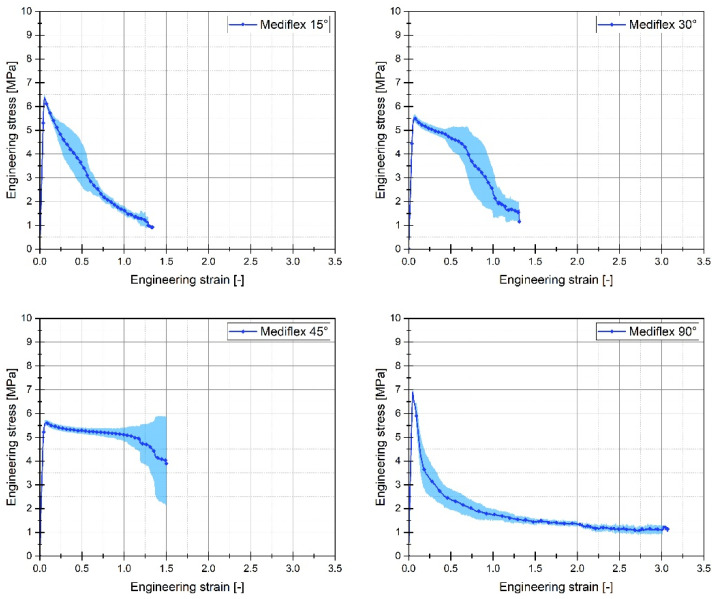
Stress-strain plots registered for 3D printed Mediflex filament with consideration of different raster angles: 15°, 30°, 45° and 90°.

**Figure 8:**
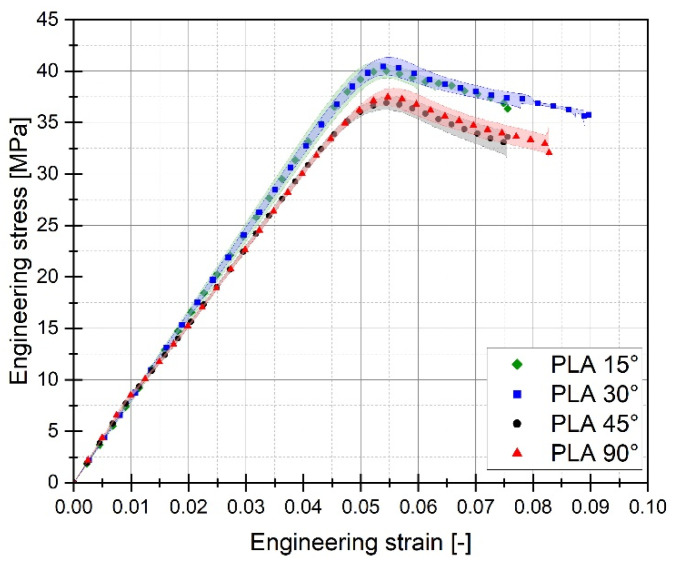
Comparison of stress-strain plots registered for 3D printed PLA PolyMax filament with consideration of different raster angles: 15°, 30°, 45° and 90°.

**Figure 9:**
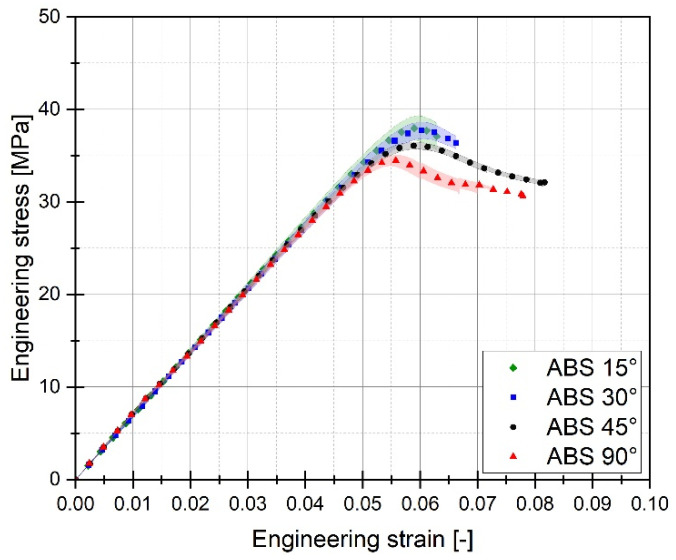
Comparison of stress-strain plots registered for 3D printed ABS Plus filament with consideration of different raster angles: 15°, 30°, 45° and 90°.

**Figure 10:**
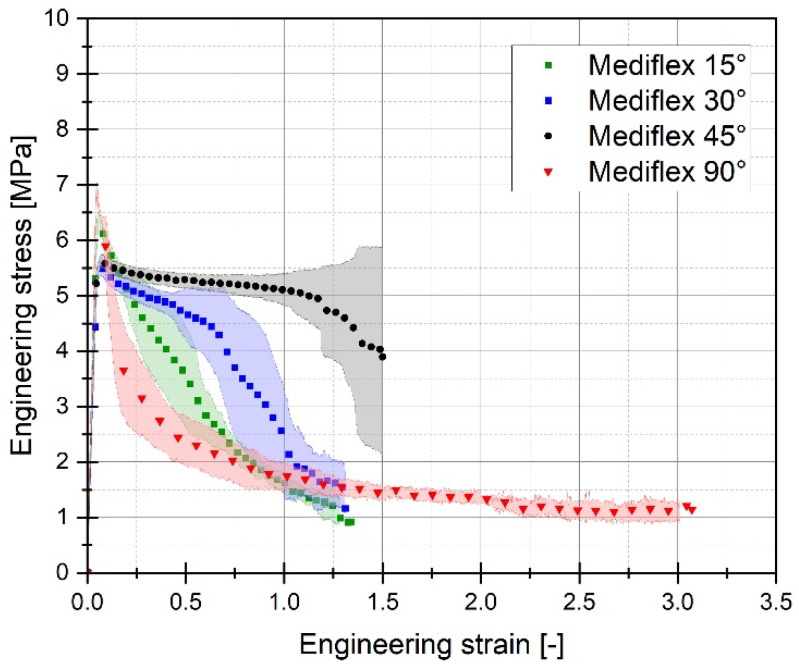
Comparison of stress-strain plots registered for 3D printed Mediflex filament with consideration of different raster angles: 15°, 30°, 45° and 90°.

For each of the considered sample filling variants, the quasi-static tensile tests were conducted six times. The graphs below (Figure 5, Figure 6 and Figure 7) present the averaged stress-strain curves along with the standard deviation. They were used to estimate the mechanical properties like Young’s Modulus, as well as tensile strength and maximum plastic strain which are presented in Table 3. Furthermore, to enable a more precise comparison of the curves obtained for the individual variants of the conducted experimental studies, additional cumulative graphs were developed, as shown in the figures. (Figure 8, Figure 9 and Figure 10).

Analysing the stress-strain curves obtained from tensile tests for various infill angles of PolyMax PLA material, one can observe a consistent reproducibility of the curves and a small standard deviation relative to the mean values. The samples with infill angles of 15 and 30 degrees exhibited the highest mechanical strength. The lowest strength was observed for the samples with a 45-degree infill angle. Similar conclusions can be drawn from the tensile test results for ABS Plus material. The maximum strength was achieved at infill angles of 15 and 30 degrees, while infill angles of 45 and 90 degrees resulted in the highest range of plastic deformation. A different deformation behaviour was observed for Mediflex material. The stress-strain curves for different proposed infill angles showed a significant variation in the range of plastic deformation. There was a noticeable standard deviation relative to the mean values, and the individual curves differed significantly in the range of plastic deformation achieved.

Based on the analysis of the data presented in Table 3, it can be stated that for the material PLA, the highest value of mechanical strength was recorded for an infill angle of 30 degrees. Additionally, for this particular variant, the range of plastic deformation of the material was also the largest. Similar observations can be made when analysing the data concerning the ABS plus material. In this case, the highest mechanical strength was noted for the sample variant with an infill angle of 15 percent, which does not differ significantly from the case where the infill angle was 30 percent. For ABS plus, the highest deformation value was recorded for the sample material variant with an infill angle of 45 degrees.

Analysing the data from the static tensile test for the Mediflex material, it can be observed that it belongs to a group of polymer materials characterised by a very large range of plastic deformation with low mechanical strength. For this material, the highest strength was determined for an infill angle of 90 degrees. Additionally, for this infill angle variant, the range of plastic deformation was twice as high as for the variant with the highest Young’s modulus (15 degrees variant). Unfortunately, despite the considerable range of deformation (up to 300%), this material exhibits a low range of mechanical strength (less than 7 MPa).

**Figure 11:**
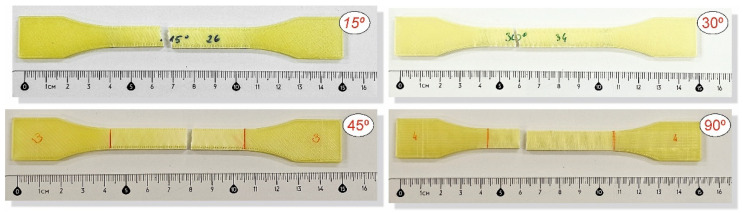
Main view of dog-bone specimens made from ABS plus after tensile tests with consideration of different raster angles: 15°, 30°, 45° and 90°.

**Figure 12:**
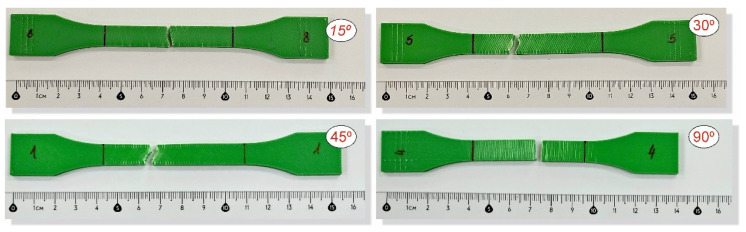
Main view of dog-bone specimens made from PLA after tensile tests with consideration of different raster angles: 15°, 30°, 45° and 90°.

**Figure 13:**
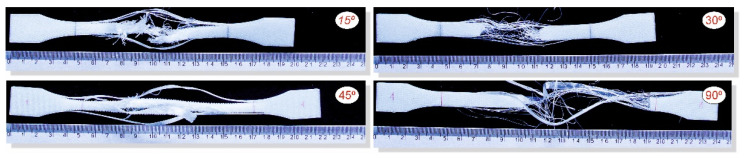
Main view of dog-bone specimens made from Mediflex after tensile tests with consideration of different raster angles: 15°, 30°, 45° and 90°.

**Figure 14:**
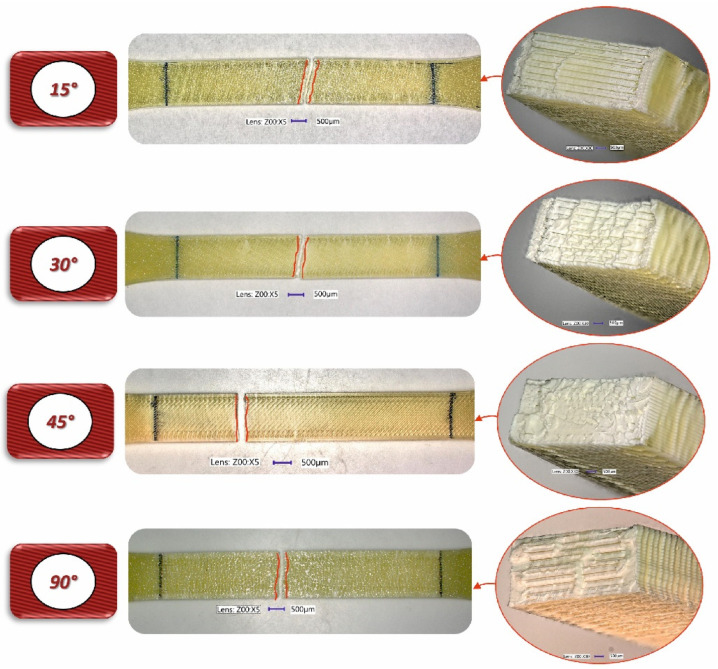
View of fracture surface of tensile specimen made from ABS plus filament with consideration of different raster angles: 15°, 30°, 45° and 90°.

**Figure 15:**
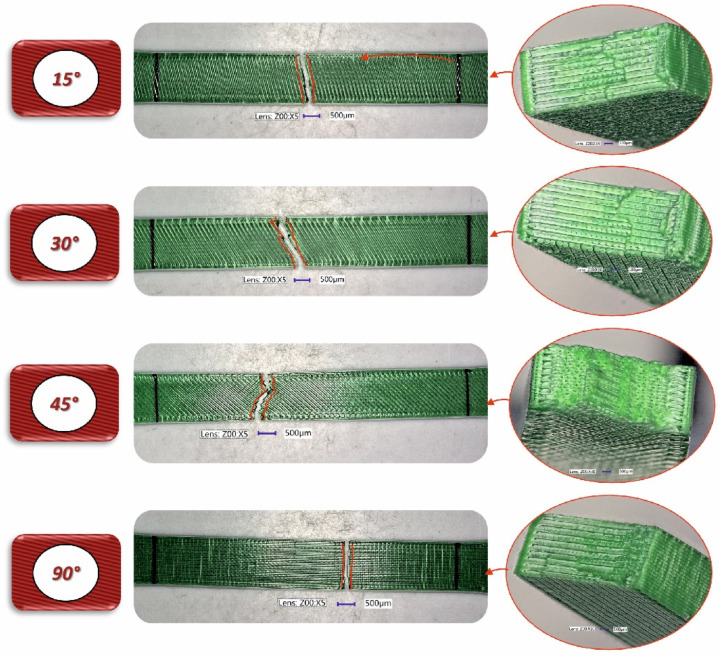
View of fracture surface of tensile specimen made from PLA filament with consideration of different raster angles: 15°, 30°, 45° and 90°.

To analyse the impact of the infill angle of the material sample on its mechanical properties more precisely, a fractographic evaluation of the samples after testing was conducted. Figure 11, Figure 12 and Figure 13 show images of the samples post-testing, taking into account the different infill angle variants. In the case of ABS plus material (Figure 11 and Figure 14), no significant differences were observed in the fracture surface pattern of the material. For all cases analysed, it has a similar linear pattern. The fracture surface pattern differs for samples made of PLA material (Figure 12 and Figure 15). It can be observed that for the infill angle variants of 15 and 90 degrees, the fracture surface is relatively flat. For infill angles of 30 and 45 degrees, the fracture surface is significantly larger.

**Figure 16:**
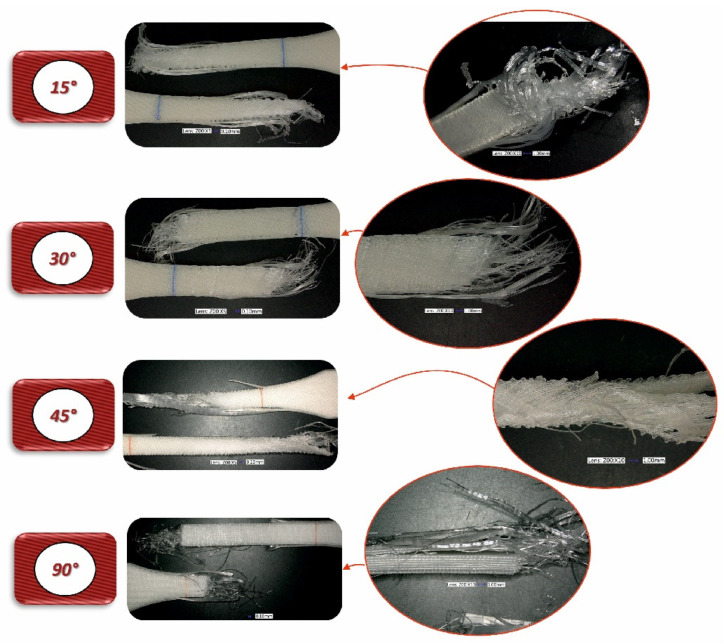
View of fracture surface of tensile specimen made from Mediflex filament with consideration of different raster angles: 15°, 30°, 45° and 90°.

In the case of assessing the fracture characteristics of samples made from Mediflex material (Figure 13 and Figure 16), it can be noted that this material exhibits similar properties to composite materials. After tensile testing, the sample was destroyed except for the side scraps. The failure mechanism pattern is similar to delamination.

The next stage of evaluating the impact of the infill angle on the mechanical strength of 3D printed material samples was conducted using a Charpy impact testing setup. In the proposed study, the influence of the printing direction during the 3D printing process was also considered. The samples were oriented horizontally and placed on their side relative to the worktable of the 3D printer.

**Figure 17:**
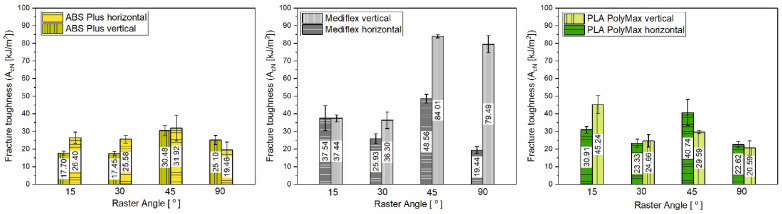
Comparison of Charpy tests results obtained for samples with different raster angle orientations (15°, 30°, 45° and 90°) and 3D printing directions (horizontal and vertical).

The results of the dynamic instrumental Charpy test are presented in Table 4 and Table 5, as well as in a graphical form in Figure 17. A slight difference in impact strength properties, according to the raster angle and building orientation, particularly in maximum overload, is observed. PLA specimens printed horizontally exhibit a peak force approximately 25% higher than those printed vertically. Additionally, PLA samples printed horizontally with a raster angle of 45° demonstrate significantly higher impact strength compared to other PLA specimens. A similar trend is observed in Mediflex specimens printed both vertically and horizontally, where the maximum force and toughness are greater for 45° raster angles.

**Figure 18:**
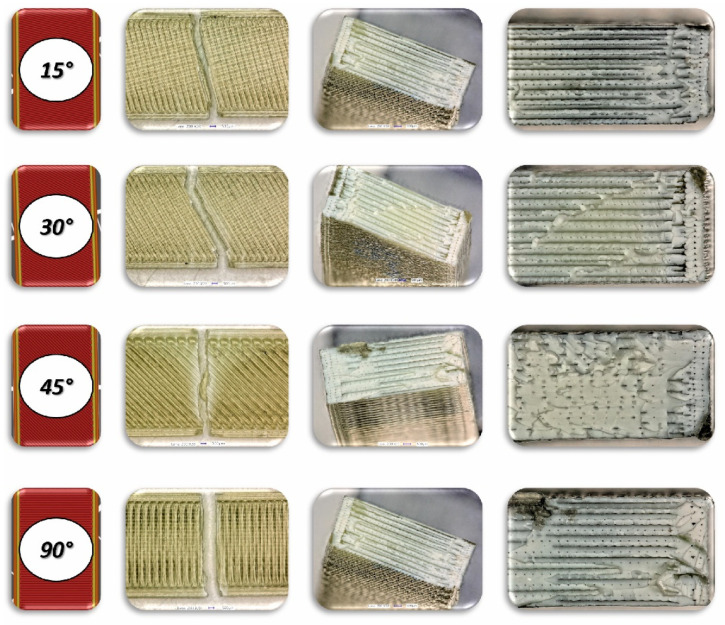
View of the fracture surface of Charpy test specimen made horizontally from ABS plus filament with consideration of different raster angles: 15°, 30°, 45° and 90°.

**Figure 19:**
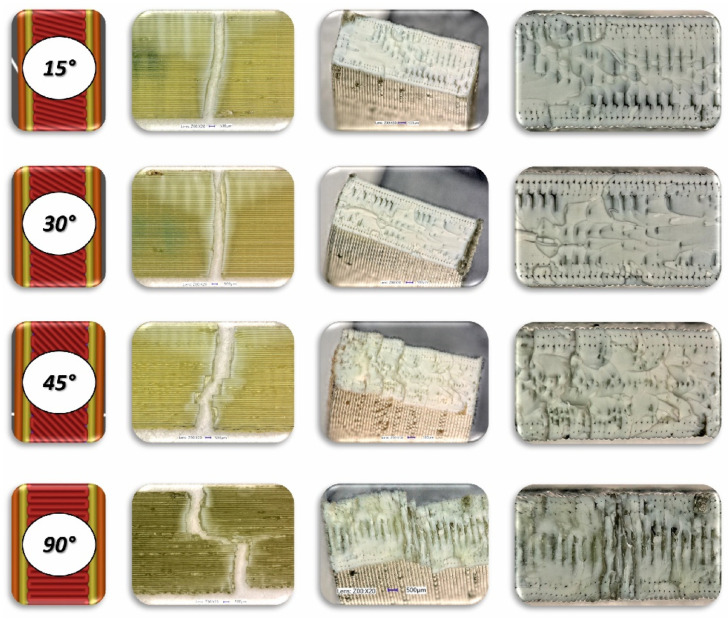
View of the fracture surface of Charpy test specimen made vertically from ABS plus filament with consideration of different raster angles: 15°, 30°, 45° and 90°.

**Figure 20:**
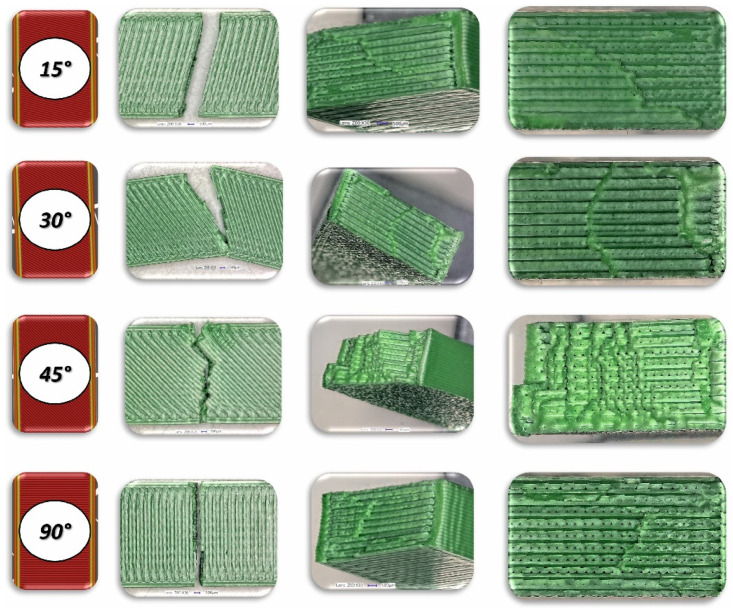
View of the fracture surface of Charpy test specimen made horizontally from PLA filament with consideration of different raster angles: 15°, 30°, 45° and 90°.

**Figure 21:**
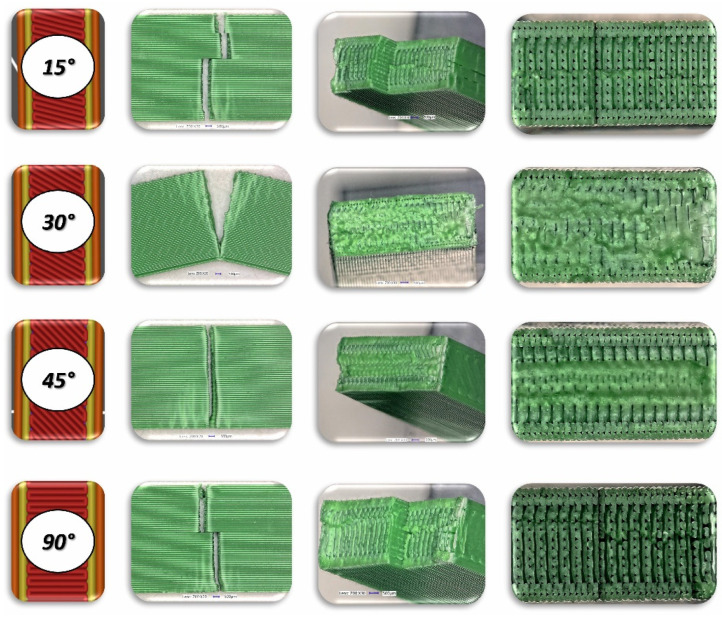
View of the fracture surface of Charpy test specimen made vertically from PLA filament with consideration of different raster angles: 15°, 30°, 45° and 90°.

**Figure 22:**
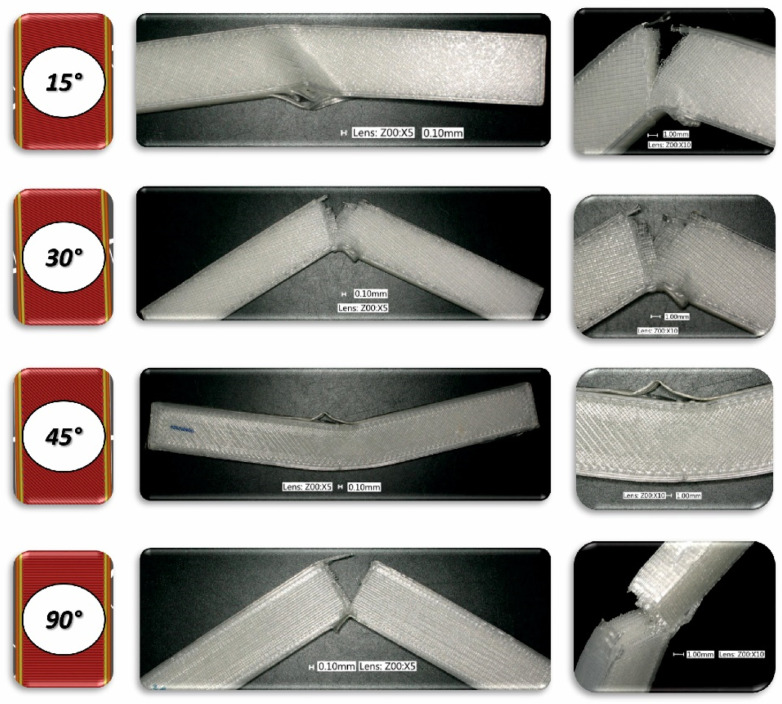
View of the fracture surface of Charpy test specimen made horizontally from Mediflex filament with consideration of different raster angles: 15°, 30°, 45° and 90°.

**Figure 23:**
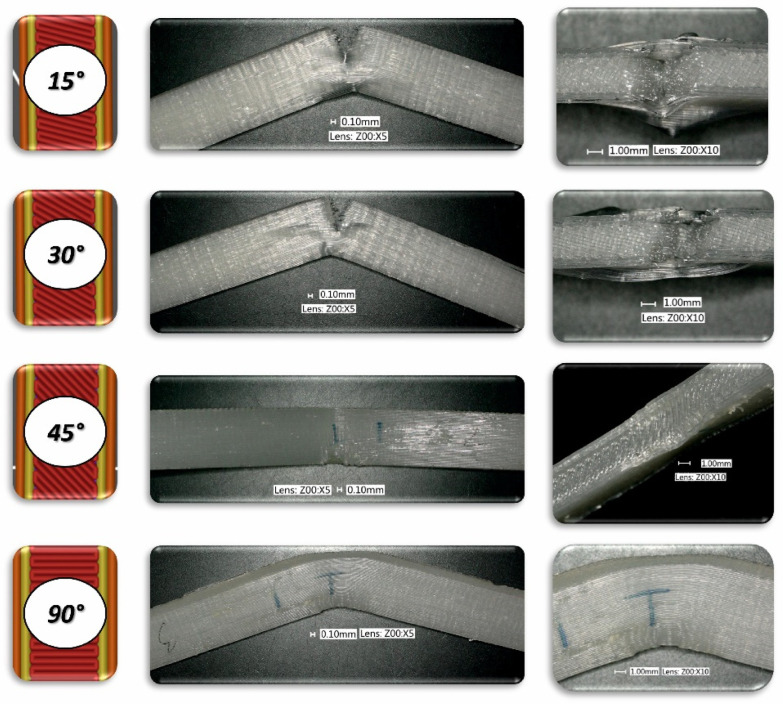
View of the fracture surface of Charpy test specimen made vertically from Mediflex filament with consideration of different raster angles: 15°, 30°, 45° and 90°.

To conduct a thorough analysis of the effect of the infill angle on the mechanical strength of additively manufactured samples under impact loading conditions, an additional fractographic assessment of the fracture surfaces of the samples after testing was performed. Using a digital microscope Keyence VHX-6000 Keyence International, Osaka, Japan, a series of photographs were taken for each of the considered infill variants, which were subsequently analysed (Figure 18, Figure 19, Figure 20, Figure 21, Figure 22 and Figure 23). The conclusions drawn from the fractographic assessment will be discussed in detail in the next chapter of the work. Nonetheless, it can be stated that the infill angle of the material sample has a significant impact on the obtained impact resistance test results.

## 4. Discussion

### Building Orientation and Raster Angle

The tested 3D printed materials exhibit similar deformation behaviour, characterized by a slightly higher elongation and lower yield stress in additively manufactured (AM) specimens with a raster angle of 45° compared to those with a 90° angle. This may be attributed to the fact that the primary load-bearing fibres in samples with a 90° angle are aligned with the force direction. Consequently, the yield point and Young’s modulus values are likely to be comparable to the filament properties provided in the datasheets by material manufacturers. In contrast, for samples with a raster angle of 45°, it is possible that voids between the successive material layers collapse during deformation until the point where the fibres start to fracture, as observed on the fracture surfaces. While most publications focusing on similar investigations, e.g., [17,20,22], present the highest mechanical response for raster angle 45°, and the lowest for 0°, Hikmat et al. [32] reported comparable findings for PLA material as are presented in this paper, where 30° has the highest ultimate tensile strength.

The differences in the influence of the raster angle on the mechanical response are particularly noticeable for the Mediflex material. Both the tensile curves and microscopic images reveal that the successive stages of deformation exhibit significant variations in material behaviour during tension for each raster angle. In the case of the 45° raster angle, Mediflex specimens appear to deform uniformly, achieving an elongation of approximately 1.52. Interestingly, the nominal stress increases slightly, which may indicate a reorientation of the fibres, with the fibres tending to align in the direction of the applied force.

For specimens with a 90° raster angle, after reaching the yield point of the Mediflex material, visible delamination occurs at the interfaces between layers, which are oriented perpendicularly to the force direction. This suggests that the adhesion between the fibres is insufficient, leading to sudden delamination, which weakens the sample and ultimately causes failure. As a result, the load is primarily carried by the fibres aligned with the force direction. Notably, despite most of the fracturing fibres, the wall fibres hold the two parts of the sample together in the final deformation stage, resulting in an elongation of 307%.

A similar phenomenon was observed by the authors of a previous study [15], who noted that elastic materials exhibit better performance when the fibre orientation aligns with the load. It is also worth mentioning that, while the yield point and elongation values are higher for the 90° raster angle, the fracture stress remains constant for samples with a 45° angle, a trend not observed in the 90° samples. The results of the impact tests of the printed samples show higher values of peak force and toughness for samples printed at a raster angle of 45° in comparison to 90° specimens. The main reason for such a phenomenon is that, during impact, the fibres have a higher mechanical resistance and the subsequent layers do not delaminate, which can be observed in the case of a 90° raster angle.

The force required to delaminate successive fibres is significantly lower than that required to fracture the material, resulting in a reduced toughness in the tested specimens. In contrast to the findings by Salim et al. [33], where ABS exhibited superior impact performance compared to PLA, the Polymax PLA material in this study demonstrated enhanced impact strength, particularly for the 45° orientation.

Regarding the flexible Mediflex material, it was observed that it possesses much higher toughness than both PLA and ABS. However, the force required to fracture Mediflex Charpy specimens is lower. Based on the morphology of the fracture surfaces, it can be concluded that, during impact, voids within the Mediflex material collapse, while in the other materials such a phenomenon was not observed. It is possible that this was the main mechanism responsible for fracture, where the presence of imperfections act as geometric notches initiating the fracture mechanism [34]. Additionally, the fracture surface morphology indicates that Mediflex specimens undergo ductile deformation, unlike the brittle failure observed in the other materials. It is also noteworthy that some Mediflex specimens bent without breaking, sliding off the supports during testing. An exception was observed for specimens with a 90° raster angle and horizontal orientation on the build platform, where approximately 80% of the samples fractured. In these cases, distinct layers were visible on the fracture surface, while for other build orientations, the fracture pattern resembled that of a solid material.

It should be noted that a comparison between the mechanical properties provided by the manufacturer and those obtained in this study reveals that the printing process conditions significantly influence the mechanical properties of additively manufactured (AM) materials. Most manufacturers either do not provide any or provide incomplete information about the printing conditions under which the mechanical properties in their datasheets were achieved. The authors of this study made several attempts to optimise printing conditions using the open Prusa 3D system for the first three materials described. Many of these attempts, especially with the Mediflex material, were unsuccessful due to challenges such as delamination, detachment from the build plate, and print jams, all of which were encountered during trial-and-error efforts to produce visually acceptable benchmark specimens. Nevertheless, the results obtained for the Mediflex material in this research differ significantly from the properties specified by the manufacturer.

## 5. Conclusions

The study presents an analysis of the influence of build orientation and raster angle on the mechanical properties of the tested specimens. ABS+, PLA PolyMax, and Mediflex filaments were selected to provide a comprehensive characterisation of additively manufactured (AM) materials with diverse mechanical properties. Specimens made from these filaments were 3D printed using the Fused Filament Fabrication (FFF) technique. To evaluate the mechanical properties of the selected AM materials, uniaxial tensile tests and non-instrumented impact drop tests were performed. Based on the experimental results, the following conclusions can be drawn:

- Build orientation, raster angle, printing settings, and environmental control (such as the presence of a heated chamber) are critical factors in achieving high-quality specimens. While build orientation primarily influences the dimensional accuracy of the specimens, raster angle and adjustable printing settings are key for ensuring strong adhesion between successive layers.
- The uniaxial tensile tests indicate that a raster angle of 45° results in greater elongation and lower yield stress in comparison to specimens printed with a raster angle of 90°, except for Mediflex specimens.
- From the impact drop tests, it was observed that regardless of build orientation, PLA and ABS specimens exhibit brittle fracture behaviour and low toughness. In contrast, Mediflex specimens primarily undergo ductile deformation and demonstrate high toughness. However, the mechanical behaviour of Mediflex depends on the raster angle and build orientation.
- The mechanical property data provided by manufacturers for these tested materials are often inaccurate or incomplete, particularly in terms of printing parameters. Therefore, the authors recommend conducting tensile tests at the very least to validate the data provided with the materials.

This study underscores the importance of optimising 3D printing parameters to accurately assess and improve the performance of AM materials.

## Figures and Tables

**Table 1:** Specification of material properties provided by filament distributors.

Material	Density [g/cm^3^]	Elastic Modulus [MPa]	Tensile Strength [MPa]	Elongation at Break [%]
PLA PolyMax ^1^	1.17–1.24	1879 ± 109	28.1 ± 1.3	1.36 ± 0.3
ABS+ ^2^	1.04	X	42.2	30
Mediflex ^3^	0.89	9.9	33	>700

^1^ PolyMax by Polymaker, ^2^ ABS+ by Devil Design, ^3^ Mediflex by Noctuo.

**Table 2:** Identified 3D printing parameters used in sample building process.

Material	Nozzle Temperature [°C]	Bed Temperature [°C]	Wall Printing Speed [mm/s]	Infill Printing Speed [mm/s]	Layer Height [mm]	Layer Width [mm]
PLA PolyMax ^1^	205	60	30	40	0.2	0.4
ABS+ ^2^	255	100	30	40	0.2	0.4
Mediflex ^3^	215	60	30	40	0.2	0.4

^1^ PolyMax by Polymaker, ^2^ ABS+ by Devil Design, ^3^ Mediflex by Noctuo.

**Table 3:** Average experimental results of tensile tests, manufactured horizontally and given accordingly.

Material	Raster Angle	E (MPa)	σ_y_ (MPa)	ε_f_ (-)
PLA PolyMax	15°	2021	40.02 ± 0.59	0.075 ± 0.006
	30°	2079	40.46 ± 0.88	0.089 ± 0.007
	45°	1867	36.92 ± 0.23	0.075 ± 0.007
	90°	1772	37.46 ± 0.34	0.082 ± 0.003
ABS+	15°	1558	37.98 ± 1.34	0.062 ± 0.002
	30°	1707	37.74 ± 0.87	0.066 ± 0.003
	45°	1869	36.09 ± 0.82	0.081 ± 0.009
	90°	1780	34.45 ± 1.59	0.078 ± 0.01
Mediflex	15°	253	6.34 ± 0.08	1.34 ± 0.11
	30°	132	5.52 ± 0.15	1.32 ± 0.17
	45°	243	5.58 ± 0.39	1.52 ± 0.52 *
	90°	145	6.83 ± 0.12	3.072 ± 0.18 *

* Elongation of non-fractured specimen for 1 MPa stress level.

**Table 4:** Toughness results of Charpy impact test for horizontally orientated samples.

Material	Raster Angle	F_max_ (N)	a_cN_ (kJ/m^2^)
PLA PolyMax	15°	637.95 ± 31.24	30.91 ± 1.91
	30°	587.35 ± 23.86	23.33 ± 2.31
	45°	583.01 ± 42.03	40.74 ± 7.47
	90°	550.41 ± 18.69	22.62 ± 1.80
ABS+	15°	447.16 ± 6.41	17.70 ± 1.17
	30°	440.93 ± 12.84	17.45 ± 1.21
	45°	534.21 ± 11.61	30.48 ± 2.94
	90°	561.13 ± 11.91	25.10 ± 2.54
Mediflex	15°	81.66 ± 3.64	37.54 ± 7.14
	30°	92.79 ± 5.52	25.93 ± 2.78
	45°	111.59 ± 2.87	48.56 ± 2.44
	90°	125.86 ± 5.24	19.44 ± 2.00

**Table 5:** Toughness results of Charpy impact test for vertically orientated samples.

Material	Raster Angle	F_max_ (N)	a_cN_ (kJ/m^2^)
PLA PolyMax	15°	689.73 ± 32.57	45.24 ± 5.08
	30°	448.92 ± 22.67	24.66 ± 3.47
	45°	518.68 ± 31.93	29.59 ± 0.96
	90°	434.10 ± 36.58	20.59 ± 4.06
ABS+	15°	505.60 ± 8.67	26.40 ± 3.21
	30°	519.22 ± 11.28	25.58 ± 2.22
	45°	598.37 ± 65.85	31.92 ± 7.24
	90°	553.78 ± 72.56	19.46 ± 4.53
Mediflex	15°	91.66 ± 2.79	37.54 ± 2.00
	30°	95.55 ± 2.72	25.93 ± 4.72
	45°	154.70 ± 6.04	84.01 ± 0.90
	90°	161.51 ± 1.20	79.49 ± 4.77

## Data Availability

The original contributions presented in this study are included in the article. Further inquiries can be directed to the corresponding author.
